# Supplementary material for: A Proof of Principle 2D Spatial Proteome Mapping Analysis Reveals Distinct Regional Differences in the Cardiac Proteome
Source: Life (Basel). 2024 Aug 1;14(8):970. doi: 10.3390/life14080970 (PMC11355120; doi:10.3390/life14080970)
Supplement: Supplementary file 1 [file life-14-00970-s001.zip › life-3007870-supplementary.pdf]

Supplementary data:

A proof of principle 2D Spatial Proteome Mapping Analysis Reveals Distinct Regional Differences in the Cardiac Proteome

Supplementary data contents:

Figure S1. Heart map distribution of cardiac cell type markers

Figure S2. Cardiac M-band protein expression

Figure S3. Correlation of citrate synthase with other common mitochondrial proteins

Figure S4. Expression of enzymes involved with fatty acid oxidation

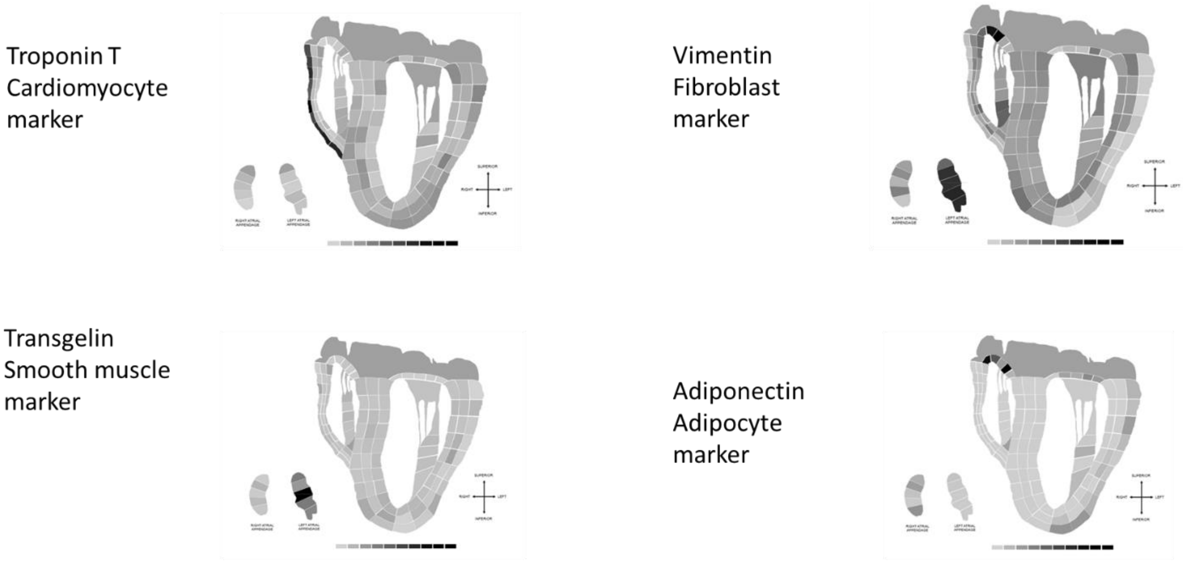

Supplementary figure S1. Heart map distribution of cardiac cell type markers

A

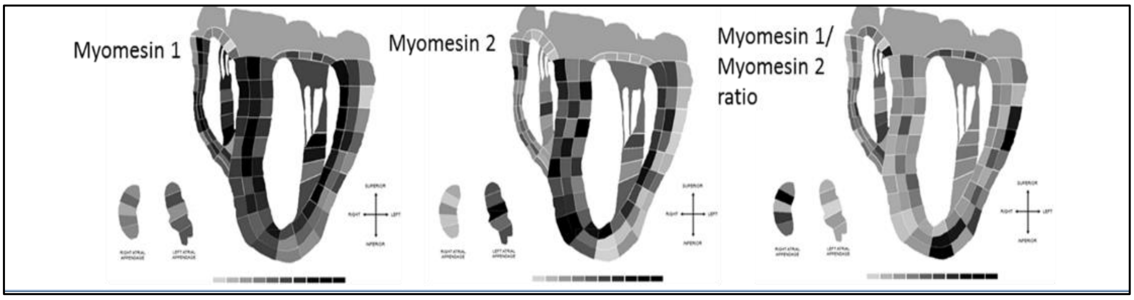

B

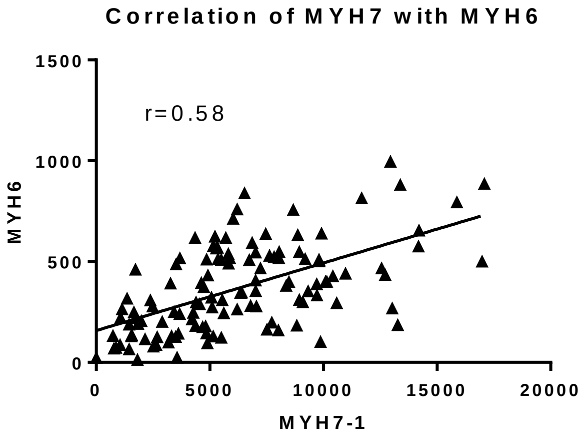

C

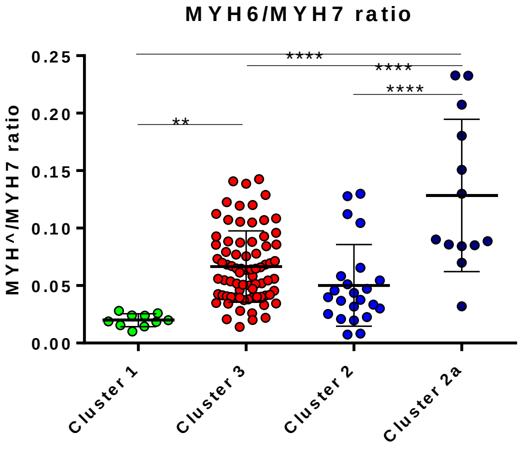

D

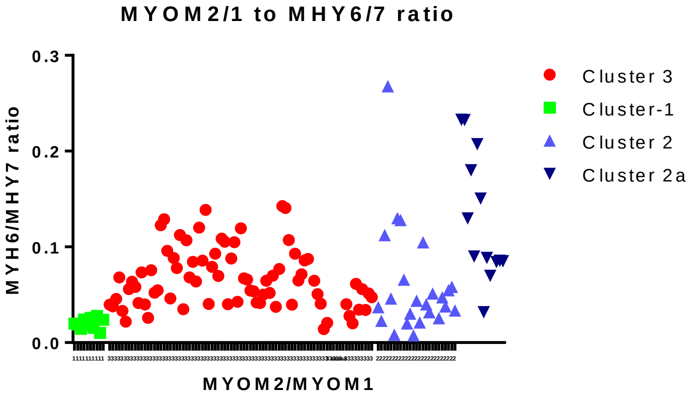

Figure S2. **Cardiac M-band protein expression**. **A** Myomesin 1 and Myosmesin 2 expression varies across the heart with reduced expression observed in the outer LV wall. However the ratio between the two indicates preferential expression of myomesin 1 and likely presecence of striated muscle in the outer LV wall. **B** Contractile protein analysis. A positive pearson correlation of unique peptides from MYH7 and MYH6 in all heart tissue sections r2= 0.58, p<0.0001. **C** Comparison of the MYH6/MYH7 ratio across the region defined clusters shows cluster has lower ratio dues to higher MYH7 expression in this region and cluster 2a

(outer RV wall) has greater ratio. Significance determined by one way ANOVA post tukey test **p<0.01, ***p<0.001,****p<0.00001 . **D**. Correlation analysis of MYH6/MYH7 ratio to MYOM2/MYOM1 ratio shows no relationship.

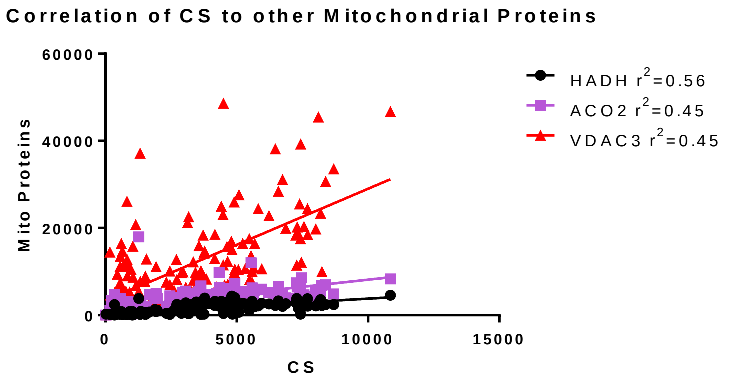

Figure S3. Correlation of citrate synthase with other common mitochondrial proteins Aconitase 2, HadH and VDAC3 to demonstrate the levels can represent mitochondrial protein.

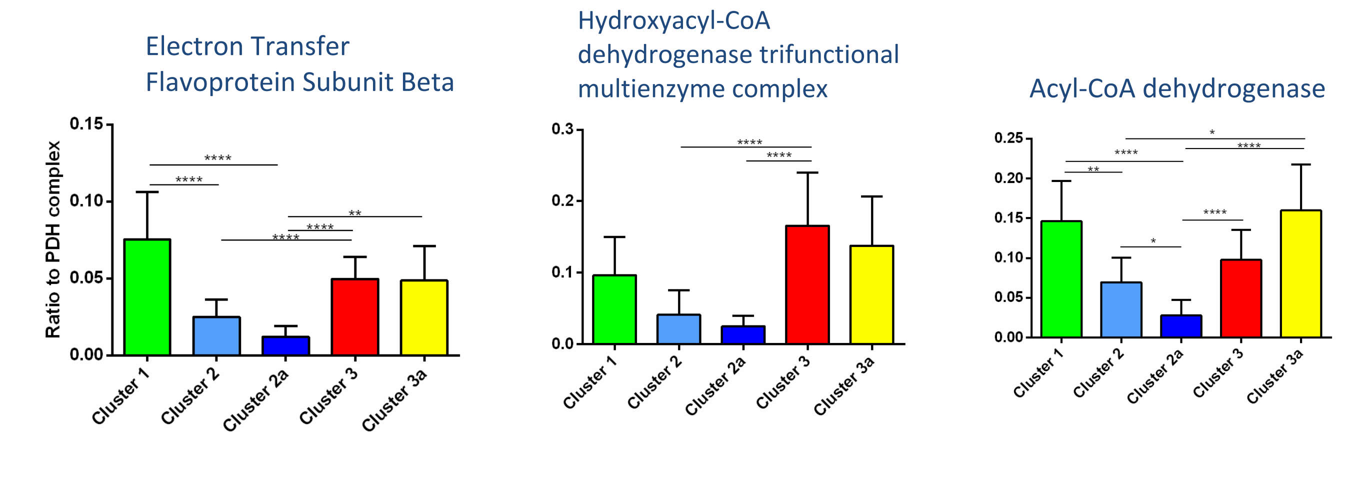

Supplementary figure S4. Expression of enzymes involved with fatty acid oxidation. Proteins are standardised to total level of proteins detected for the pyruvate dehydrogenase complex (PDHA1, PDHB, DLAT, DLD, PDHX). P values are calculated using Kruskal wallis one way ANOVA with post correction. * indicates p>0.05, ** p>0.01, **** p<0.0001
